# The host genes influencing *Clostridioides difficile* infection and the potential role of intestinal Lactobacillus acidophilus: a Mendelian randomization and animal model study

**DOI:** 10.3389/fcimb.2025.1607476

**Published:** 2025-07-08

**Authors:** Yuxin Sun, Wenzhen Zhou, Senlin Ma, Qiuxin Lu, Yinuo Yuan, Yanchao Zheng, Yifan Yang, Kangshuai Zhou, Qingjiang Chen, Gonghao Sun, Zhaoming Shang, Junwei Qian, Xiaofei Jiang, Mingquan Chen

**Affiliations:** ^1^ Department of Emergency, Huashan Hospital, Fudan University, Shanghai, China; ^2^ Department of Cardiology, Huashan Hospital, Fudan University, Shanghai, China; ^3^ Department of Infectious Diseases, Shanghai Key Laboratory of Infectious Diseases and Biosafety Emergency Response, National Medical Center for Infectious Diseases, Huashan Hospital, Fudan University, Shanghai, China

**Keywords:** *Clostridioides difficile* infection, gut probiotics, lactobacillus acidophilus, THOC5 gene, Mendelian randomization

## Abstract

**Introduction:**

*Clostridioides difficile* infection (CDI) poses a significant clinical burden due to its high recurrence rate and life-threatening complications. While gut dysbiosis is central to CDI pathogenesis, mechanisms underlying microbiota-mediated host defense remain underexplored.

**Methods:**

This study integrated summary-data-based Mendelian randomization (SMR) of cis-expression quantitative trait loci (cis-eQTLs) from blood, transverse colon, and sigmoid colon tissues with CDI genome-wide association study (GWAS) data to identify host genes influencing CDI susceptibility. Bayesian co-localization was employed to validate relationships. Then a germ-free (GF) mice model colonized with *Lactobacillus acidophilus* (LA) was used to investigate LA-mediated regulation of possible gene expression and phenotypic changes in the host.

**Results:**

SMR analysis identified 14 genes associated with CDI risk, primarily clustered in the major histocompatibility complex (MHC) region. Notably, THOC5 exhibited robust associations (P_SMR_ < 0.05 in all tissues) and co-localization evidence (posterior probability = 82.6%). In GF mice, LA colonization significantly upregulated colonic *Thoc5* expression in two independent experiments (fold change = 5.19/5.00, P = 0.034/0.031). Subsequent immunofluorescence experiments revealed that LA colonisation enhanced macrophage activation in the colonic tissue.

**Discussion:**

These findings reveal key host genes, particularly THOC5, that influence susceptibility to CDI, providing new targets for future prevention and treatment research. Additionally, the study suggests a potential mechanism by which host intestinal LA protects against CDI, highlighting the interaction between probiotics and host transcriptional networks in CDI resistance. These insights offer valuable directions for further investigation.

## 1 Introduction

*Clostridioides difficile* infection (CDI) is the primary cause of antibiotic-associated pseudomembranous colitis, accounting for 15% of healthcare-associated infections (HAIs) (Burnham and Carroll, 2013; Leffler and Lamont, 2015). Its high recurrence rate (25%) and life-threatening complications (e.g., fulminant colitis) impose a significant clinical burden (Louie et al., 2011; Kelly, 2012; Magill et al., 2018; Guh et al., 2020). As one of the first formally recognized microbiome-associated diseases (Bartlett et al., 1977), CDI pathogenesis is closely linked to gut dysbiosis. A healthy gut microbiota resists CDI through niche competition and bile acid metabolism (Surawicz and McFarland, 1999; Sorg and Sonenshein, 2008; Theriot et al., 2014; Buffie et al., 2015). Consequently, faecal microbiota transplantation (FMT) has emerged as a critical intervention for refractory CDI. Compared to conventional antibiotic therapies (e.g., vancomycin), FMT reduces recurrence rates while preventing antibiotic-associated toxicity (Drekonja et al., 2015).

However, which specific probiotic taxa within the gut microbiota confer the principal protection against CDI, and whether individual microbes exert this effect by modulating host gene transcription, remain unclear. Most published work has focused on direct microbe–microbe interactions, whereas microbiota-induced host responses are comparatively understudied (Martinez et al., 2022). Only a handful of studies have shown that commensal bacteria safeguard mucosal immunity and barrier integrity by regulating host gene expression (Kamada et al., 2013; Leclercq et al., 2014).

Among probiotics, *Lactobacillus acidophilus* (LA) has received considerable attention. Several LA-containing formulations have demonstrated anti-CDI activity and are used clinically. For instance, a proprietary product comprising LA CL1285, *L. casei* LBC80R, and *L. rhamnosus* CLR2 (Bio-K +) reduced CDI incidence in a randomized controlled trial (RCT) in a clear dose-dependent manner (Gao et al., 2010). Two additional RCTs showed that capsules containing LA NCFM (ATCC 700396) markedly alleviated CDI symptoms and shortened disease duration (Barker et al., 2017; De Wolfe et al., 2018). A meta-analysis of multiple RCTs provides sufficient evidence to recommend *Lactobacillus* species (LA and *L. casei*) for CDI prophylaxis (Wu et al., 2013). In animals and *in vitro*, LA monotherapy suppresses *C. difficile* growth and intestinal pathological injury in mice (Kaur et al., 2011; Yun et al., 2014), and diverse LA strains (including CL1285 and several ATCC isolates) exhibit direct anti-cytotoxicity activity *in vitro* (Auclair et al., 2015).

Mendelian randomization (MR) is a scientific methodology that leverages genetic variants as instrumental variables to establish causal relationships between exposures and outcomes (Greenland, 2018). Due to the random distribution of alleles and resistance to common confounding factors, MR-derived causal inferences are generally considered robust (Burgess et al., 2013). In this study, we conducted a summary-data-based Mendelian randomization (SMR) analysis (Zhu et al., 2016) using cis-expression quantitative trait loci (cis-eQTLs) from human blood, transverse colon, and sigmoid colon tissues, combined with CDI genome-wide association study (GWAS) data. This approach identified key genes whose expression is causally associated with CDI pathogenesis. Bayesian co-localisation analysis was used to determine whether the key genes identified in the SMR screen and susceptibility to CDI share a common causal variant. We then confirmed probiotic-driven gene-expression changes and their phenotypic consequences in germ-free (GF) mice mono-colonised with LA, thereby elucidating host–microbiota interaction mechanisms that may underlie defence against CDI.

## 2 Materials and methods

### 2.1 Study design

**Figure 1:**
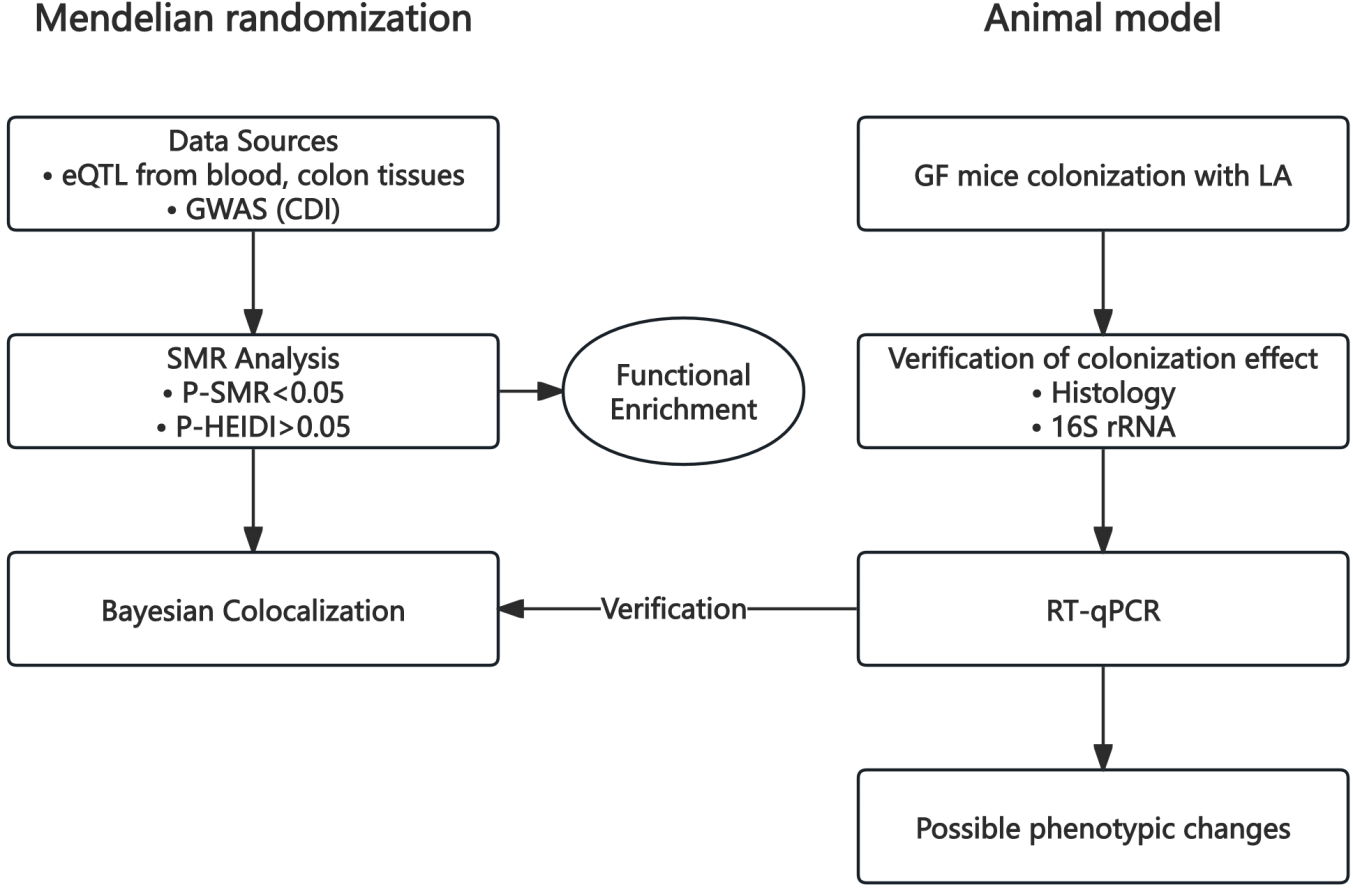
Research process diagram.

In this study, we performed a summary-data-based Mendelian randomization (SMR) analysis using cis-expression quantitative trait loci (cis-eQTLs) data from three human tissues—blood, transverse colon, and sigmoid colon—alongside genome-wide association study (GWAS) data for CDI. Subsequently, Bayesian co-localization analysis (coloc) was conducted using blood cis-eQTL data from an independent database and CDI GWAS data. All eQTL and GWAS datasets were derived from previously published studies or publicly available summary statistics provided by consortia. These studies had obtained approval from their respective Institutional Review Boards (IRBs); thus, no additional ethical review was required. Animal experiments employed a GF mouse model colonized with LA. Successful colonization was verified by crypt-depth histology and 16S rRNA profiling. RT-qPCR assessed expression changes in key genes identified by SMR and co-localisation analyses, and relevant phenotypic read-outs were recorded. Animal experiments were approved by the Ethics Committee of the Experimental Animal Center at Fudan University (Approval No. 2025-HSYY-156). A schematic overview of the workflow is shown in **Figure 1**.

### 2.2 Data sources

For the SMR analysis, cis-eQTL summary statistics for blood were obtained from the CAGE study (Lloyd-Jones et al., 2017), which investigated transcript-level gene expression in peripheral blood from 2,765 individuals of predominantly European ancestry. Cis-eQTL data for the transverse colon and sigmoid colon were sourced from the Genotype-Tissue Expression (GTEx) project (Consortium et al., 2020). The GTEx study analysed 15,201 RNA-sequencing samples derived from 49 tissues of 838 postmortem donors (primarily European Americans), including 368 transverse colon and 318 sigmoid colon tissue samples.Bayesian co-localization analysis utilized cis-eQTL data from the eQTLGen Consortium (Vosa et al., 2021), which aggregates 37 datasets comprising 31,684 blood samples.

The CDI GWAS data were obtained from the FinnGen study (Kurki et al., 2023), a large-scale genomics initiative that integrates genetic variation and health records from over 500,000 Finnish biobank participants to investigate disease mechanisms and predispositions. FinnGen is a collaborative effort between Finnish research institutions, biobanks, and international industry partners. Cases were defined as patients with *Clostridioides difficile*-induced enterocolitis, totalling 3,384 cases and 406,048 controls.

### 2.3 Summary-data-based mendelian randomization

SMR is a summary-level Mendelian randomisation framework that uses expression quantitative trait loci (eQTLs) as instrumental variables (IVs) to test the causal impact of tissue-specific gene expression on complex traits or disease risk (Zhu et al., 2016). The primary SMR test employs the most significant eQTL (topSNP)—located within ±2000 base pairs (bp) of the target gene and achieving genome-wide significance (P < 5 × 10^−8^)—to infer a causal relationship between expression and phenotype. Like conventional MR, SMR rests on three assumptions that minimise population stratification and confounding (Relton and Davey Smith, 2012): (i) the SNP is strongly associated with the exposure; (ii) the SNP is independent of confounders; and (iii) the SNP affects the outcome solely through the exposure. Adhering to these assumptions reduces vulnerability to reverse causation and residual confounding relative to observational studies.

We utilized single nucleotide polymorphisms (SNPs) from cis-eQTL data of three human tissues—blood, transverse colon, and sigmoid colon—as instrumental variables (IVs), with gene expression as the exposure and CDI as the outcome. SMR analysis was employed to integrate GWAS and eQTL summary statistics to test for pleiotropic associations between gene expression and CDI, driven by shared and potentially causal genetic variants at specific loci. The heterogeneity in dependent instruments (HEIDI) test was applied to exclude associations likely caused by high linkage disequilibrium (LD) between distinct genetic variants, requiring the P_HEIDI_ > 0.05. Default SMR settings were adopted, including: P_eQTL_  < 5 × 10 ^–8^, minor allele frequency (MAF) > 0.01, exclusion of SNPs in strong LD (r ^2^  > 0.9) with the top-associated eQTL, removal of SNPs in weak or no LD (r ^2^  < 0.05) with the top-associated eQTL, and cis-eQTLs located within ±2000 bp of each probe were selected for the SMR analysis) (Zhu et al., 2016). The P_SMR_ were adjusted for false discovery rate (FDR) using the Benjamini-Hochberg method, and genes with q-value < 0.2 were selected as key candidates.

### 2.4 The functional enrichment analysis of key genes

Functional enrichment analysis was performed on key genes identified across all three tissues. Enrichment analysis included Gene Ontology (GO) Biological Processes (BP), Cellular Components (CC), Molecular Functions (MF), and KEGG pathways. False discovery rate (FDR) correction was applied using the Benjamini-Hochberg method, with a significance threshold set at q-value < 0.05. The top 10 most significant GO terms and the top 20 most significant KEGG pathways were visualized to highlight key biological mechanisms.

### 2.5 Bayesian co-localization analysis

Bayesian co-localization analysis was conducted using the coloc method (Wallace, 2021). For each key gene, blood cis-eQTL data from the eQTLGen Consortium were co-localized with CDI GWAS data. The coloc.abf() function was applied to all variants within a ±1 megabase (Mb) region around the top-associated SNP (topSNP) in the eQTL data and the corresponding region in the disease GWAS data. The posterior probability (PP.H4) of shared causal variants between gene expression and CDI was calculated, with PP.H4 > 50% considered robust evidence for co-localization (Huang et al., 2023).

### 2.6 Animal source and grouping

GF and specific pathogen-free (SPF) male C57BL/6 mice (6 weeks old) were obtained from Slac Laboratory (Shanghai, China). GF mice were housed in sterile isolators, with weekly verification of their GF status via microscopy and aerobic/anaerobic culturing of fresh faecal samples. All GF mice received autoclaved water and a gamma-irradiated (50 kGy) standard diet. Mice were maintained at 20–24°C under a 12-hour light/dark cycle (lights on at 07:30) with 55–65% humidity. Two independent experiments were conducted with LA strains from different suppliers and distinct mouse batches. Experiment 1 used 13 mice: 5 GF mice colonised with LA (experimental), 4 GF mice gavaged with phosphate-buffered saline (PBS, negative control), and 4 SPF mice gavaged with PBS (positive control). Experiment 2 followed the identical design with 12 mice (4 per group).

### 2.7 Animal handling and sample collection

Bacteria were cultured for 24 h at 37°C in MRS broth (Hopebio, Qingdao, China) under anaerobic conditions (5% H_2_, 10% CO_2_, 85% N_2_). Bacterial cells were harvested by centrifugation at 3,000 × g for 15 minutes, washed, and resuspended in sterile PBS containing 0.1% peptone. GF mice received a daily oral gavage of 1 × 10^9^ colony-forming units (CFU) in 200 μL PBS for 14 consecutive days. On day 15, all mice were euthanized via cervical dislocation under isoflurane anesthesia. Colon tissues and luminal contents were immediately collected for downstream analyses.

### 2.8 Quantitative real-time polymerase chain reaction

Approximately 30 mg of freshly excised colon tissue was rinsed twice in PBS, freeze-dried, and homogenised in 600 µL of lysis buffer from the Cell/Tissue Total RNA Kit (NCM Biotech) using an electric homogeniser. After centrifugation, the supernatant was processed according to the manufacturer’s protocol to isolate total RNA, which was reverse-transcribed with a cDNA synthesis kit (Monad Biotech). Quantitative PCR was then carried out with the RT-PCR kit (Selleck) as per the kit instructions: each reaction contained 1 µL of forward primer and 1 µL of reverse primer (10 µmol/L each), 120 ng of template cDNA, and nuclease-free water to a final volume of 20 µL. The *Thoc5* primers (Guria et al., 2011) were as follows: forward: 5′- TCTGCCTTTTCACCTGGAAG -3′, reverse: 5′- CTCGGTACTTTTCTGCCAGC -3′. The β-actin reference gene primers (Kong et al., 2023) were: forward: 5′- AGAAGATCTGGCACCACACC -3′, reverse: 5′- TACGACCAGAGGCATACAGG -3′. Thermal cycling conditions included an initial denaturation at 95°C for 30 sec, followed by 40 cycles of denaturation at 95°C for 15 sec, annealing/extension at 60°C for 30 sec, and a final extension at 72°C for 30 sec. Relative *Thoc5* expression levels were normalized to β-actin and calculated using the 2-ΔΔCT method.

### 2.9 Histological analysis

A 2 cm segment of colon was excised from each mouse and gently rinsed with PBS at low speed and low pressure. Tissues were then fixed in 4% (wt/vol) paraformaldehyde, and embedded in paraffin. Longitudinal sections (4 μm thickness) were stained with hematoxylin (Vector Laboratories, Burlingame, CA, USA) and eosin (Sigma-Aldrich, Zwijndrecht, Netherlands). Images were captured using a digital microscope (100×), and crypt depth was measured in 10 well-preserved crypts per mouse. For the immunofluorescence (IF) study, colon sections from the first experiment were analysed (n = 3 mice per group). Sections were incubated with a primary anti-F4/80 antibody (Cell Signaling Technology, 1:500) followed by an anti-rabbit IgG secondary antibody (Jackson, 1:500). Fluorescent labelling was developed with the TYR-520 fluorophore, TSA+ signal amplifier, DAPI nuclear counterstain, and an antifade mounting medium supplied in the IF kit (Huilanbio). Slides were scanned on a fluorescence microscope, and images were captured at ×400 total magnification. Three random fields per animal were photographed, and the mean fluorescence intensity of F4/80 was quantified in Fiji. Crypt-depth measurements and IF quantification were performed independently by two pathologists who were blinded to the group assignments.

### 2.10 16S rRNA gene sequencing

Genomic DNA was extracted from mouse intestinal luminal content samples and assessed for quality via 1% agarose gel electrophoresis. Target regions were amplified using barcoded primers (TransStart FastPfu DNA Polymerase, ABI GeneAmp^®^ 9700 PCR System) under low-cycle PCR conditions, with triplicate reactions pooled and purified for each sample. Amplification products were quantified using the Quant-iT™ PicoGreen fluorescence assay (Promega, USA), and libraries were constructed with the TruSeq™ DNA Sample Prep Kit (Illumina, USA). Paired-end sequencing was performed on the NextSeq platform (Illumina, USA).

Bioinformatics analysis followed the QIIME2 pipeline: raw data underwent quality filtering, paired-end read merging, and denoising via DADA2 to generate amplicon sequence variants (ASVs). Taxonomic annotation was performed using the SILVA database. Alpha diversity indices (Shannon, Simpson, Chao1) and beta diversity metrics (Bray-Curtis, UniFrac distances) were calculated. Group differences were assessed using ANOSIM/PERMANOVA tests. Community structure was visualized via bar plots, heatmaps, and Venn diagrams. Differential taxa analysis was conducted using LEfSe and MaAsLin2 (adjusted for confounders). Functional profiling of microbial communities was inferred using PICRUSt2 and Tax4Fun, while microbial phenotypes were evaluated via BugBase.

### 2.11 Statistical methods

Statistical analyses were performed using R (version 4.3.1) and GraphPad Prism (version 9.0). Functional enrichment analysis was conducted with the clusterProfiler R package (version 4.10.0) (Wu et al., 2021). Bayesian co-localization analysis was implemented using the coloc R package (version 5.2.3) (Wallace, 2021). SMR was performed with the SMR software tool (https://yanglab.westlake.edu.cn/software/smr/#Overview) (Zhu et al., 2016). RT-qPCR data, crypt-depth measurements, and IF intensities were analysed in GraphPad Prism. Variance homogeneity was checked by one-way ANOVA, followed by Welch’s t-test where appropriate. Processing of 16S rRNA sequencing data utilized the vegan (version 2.6-4) and phyloseq (version 1.46.0) R packages. Visualization of results was performed using the ggplot2 R package (version 3.4.4). The code used in the study can be found at https://github.com/syxdavid/Bayesian-co-localization-analysis.

## 3 Results

### 3.1 SMR analysis of eQTL data and CDI

**Figure 2:**
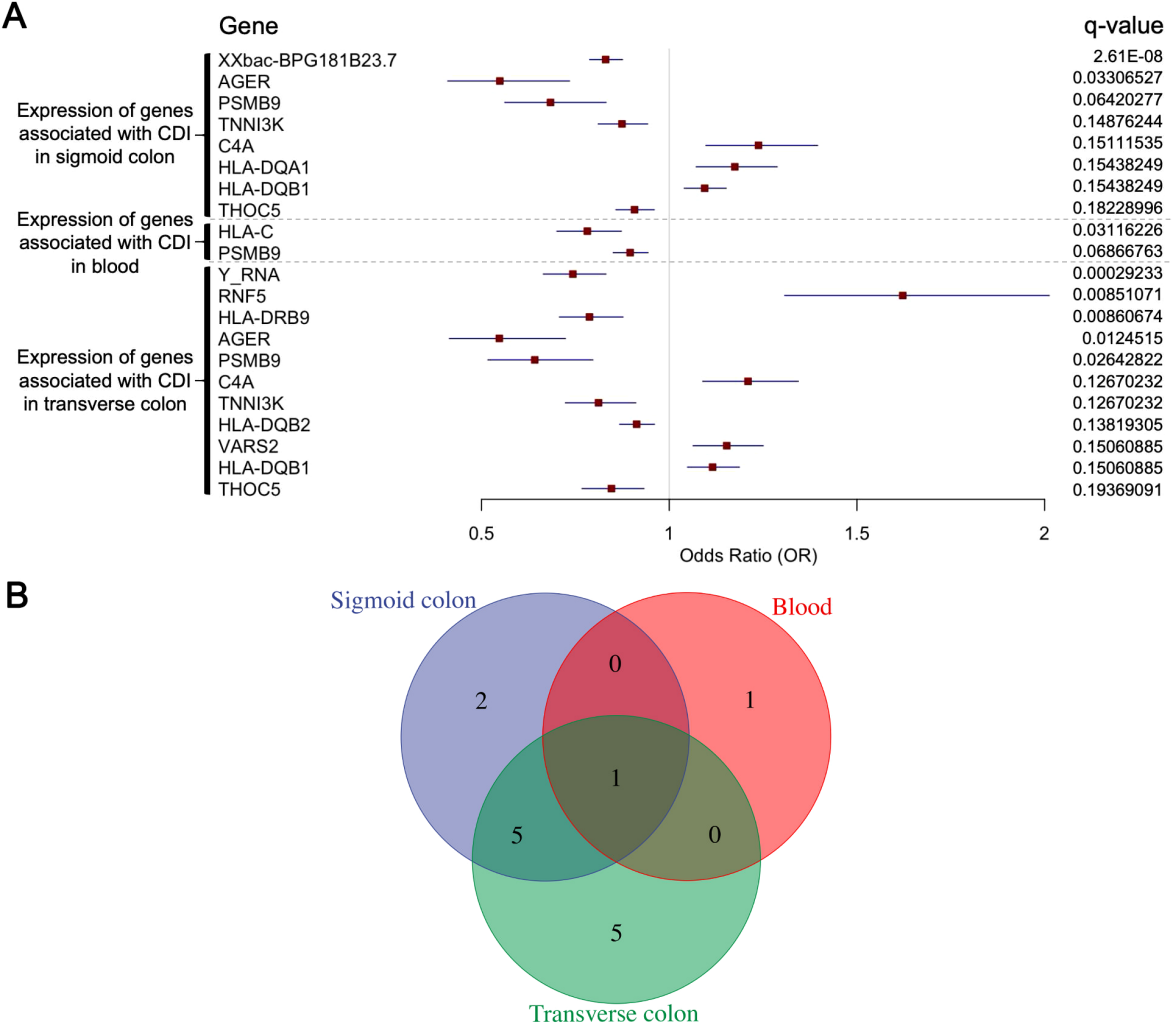
**(A)** Summary-data-based Mendelian randomization (SMR) analysis results for 14 genes, integrating cis-eQTL data with CDI GWAS data. Shown are: gene names, eQTL data sources (blood, transverse colon, sigmoid colon), odds ratio (OR), 95% confidence interval (CI), FDR-corrected q-value. **(B)** The number of genes whose expression is associated with CDI susceptibility in blood, sigmoid colon, and transverse colon, including those co-associated across two or all three tissues.

We performed SMR analysis using gene expression quantitative trait loci (eQTL) data from three tissues—blood, transverse colon, and sigmoid colon—integrated with CDI GWAS data. Genes meeting the significance threshold (P_SMR_ < 0.05) and passing the heterogeneity (HEIDI) test (P_HEIDI_ > 0.05) numbered 365, 223, and 203 in blood, transverse colon, and sigmoid colon tissues, respectively (**Supplementary Table 1**). We detected 14 key genes across the three tissues analysed (**Figure 2A**).

Sigmoid colon: XXbac-BPG181B23.7 (OR = 0.83, 95% CI 0.79–0.88; P_SMR_ = 5.36 × 10^−12^; q = 2.61 × 10^−8^), AGER (0.55, 0.41–0.73; 5.43 × 10^−5^; 3.31 × 10^−2^), PSMB9 (0.68, 0.56–0.83; 1.45 × 10^−4^; 6.42 × 10^−2^), TNNI3K (0.87, 0.81–0.94; 4.58 × 10^−4^; 1.49 × 10^−1^), C4A (1.24, 1.10–1.40; 4.96 × 10^−4^; 1.51 × 10^−1^), HLA-DQA1 (1.17, 1.07–1.29; 5.78 × 10^−4^; 1.54 × 10^−1^), HLA-DQB1 (1.09, 1.04–1.15; 5.52 × 10^−4^; 1.54 × 10^−1^), THOC5 (0.91, 0.86–0.96; 7.48 × 10^−4^; 1.82 × 10^−1^).

Transverse colon: Y RNA (0.74, 0.66–0.83; 2.15 × 10^−7^; 2.92 × 10^−4^), RNF5 (1.62, 1.31–2.01; 1.10 × 10^−5^; 8.51 × 10^−3^), HLA-DRB9 (0.79, 0.71–0.88; 1.33 × 10^−5^; 8.61 × 10^−3^), AGER (0.55, 0.41–0.72; 2.29 × 10^−5^; 1.25 × 10^−2^), PSMB9 (0.64, 0.52–0.80; 5.84 × 10^−5^; 2.64 × 10^−2^), C4A (1.21, 1.09–1.34; 3.82 × 10^−4^; 1.27 × 10^−1^), TNNI3K (0.81, 0.72–0.91; 3.97 × 10^−4^; 1.27 × 10^−1^), HLA-DQB2 (0.91, 0.87–0.96; 4.58 × 10^−4^; 1.38 × 10^−1^), VARS2 (1.15, 1.06–1.25; 5.54 × 10^−4^; 1.51 × 10^−1^), HLA-DQB1 (1.12, 1.05–1.19; 5.55 × 10^−4^; 1.51 × 10^−1^), THOC5 (0.85, 0.77–0.93; 7.91 × 10^−4^; 1.94 × 10^−1^).

Blood: HLA-C (0.78, 0.70–0.87; 1.10 × 10^−5^; 3.12 × 10^−2^), and PSMB9 (0.90, 0.85–0.94; 3.23 × 10^−5^; 6.87 × 10^−2^).

Of these, AGER, TNNI3K, C4A, HLA-DQB1, and THOC5 were significant in two tissues, while PSMB9 reached significance in all three (**Figure 2B**). Notably, most of these genes clustered within the major histocompatibility complex (MHC) region on chromosome 6 (29–33 Mb) (D’Antonio et al., 2019; Douillard et al., 2021), with their top-associated SNPs localized to adjacent genomic positions.

### 3.2 Functional enrichment analysis of key genes

After excluding duplicate and unannotated genes, 12 key genes were subjected to functional enrichment analysis. Gene Ontology (GO) analysis revealed 30 significantly enriched terms (q-value < 0.05), highlighting antigen binding, processing, and presentation, MHC class II molecule activity, and immune regulation (**Supplementary Figure 11A**). KEGG pathway analysis identified 20 enriched pathways (q-value < 0.05), including T-cell differentiation, inflammatory/autoimmune diseases, infectious diseases, and pathogen clearance (**Supplementary Figure 11B**).

### 3.3 Bayesian co-localization analysis

**Figure 3:**
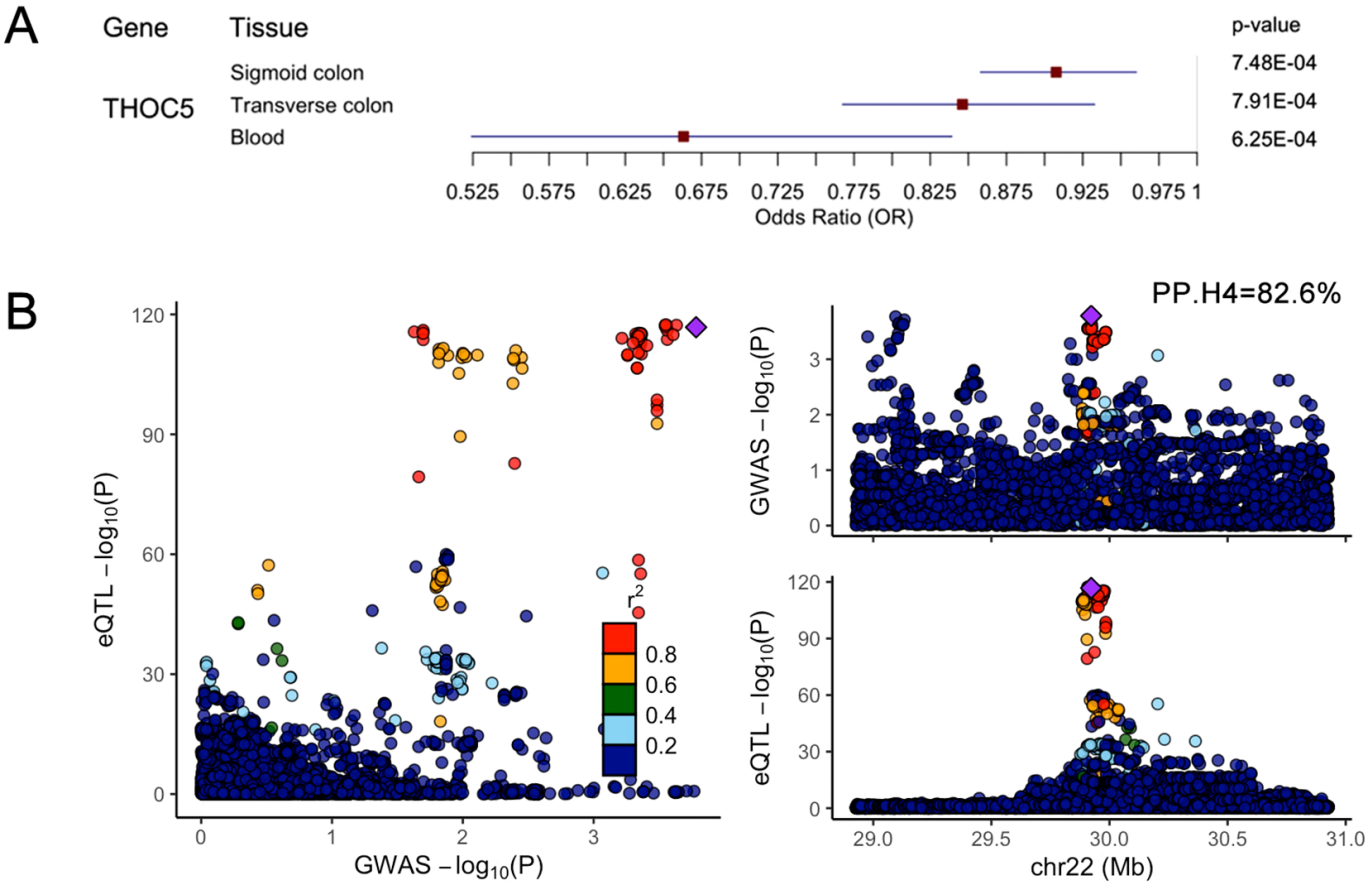
**(A)** SMR analysis results for THOC5 eQTL data across three tissues (blood, transverse colon, sigmoid colon) and CDI GWAS data. Included are: eQTL data sources, odds ratio (OR), 95% confidence interval (CI), P_SMR_. **(B)** Bayesian co-localisation of the THOC5 cis-eQTL signal with the CDI GWAS locus. Each dot represents a single SNP. The x-axis (GWAS –log_10_P) denotes the significance of association between that SNP and susceptibility to CDI, while the y-axis (eQTL –log_10_P) shows the significance of association between the same SNP and THOC5 expression. The purple diamond marks the lead GWAS SNP on chromosome 22, used as the index variant for linkage-disequilibrium (LD) calculations; r² values indicate the degree of LD between each SNP and the lead SNP. The posterior probability for hypothesis H4—that THOC5 expression and CDI susceptibility share a single causal variant—is 82.6%, supporting a common underlying signal.

Bayesian co-localization analysis of key gene eQTLs (from the eQTLGen Consortium) and CDI GWAS data revealed robust evidence for shared causal variants only at the THOC5 locus (posterior probability, PP.H4 = 82.6%; **Figure 3B**). Results for other genes are provided in **Supplementary Figures 1–10**. THOC5 exhibited significant SMR associations (P_SMR_ < 0.05) across all three tissues, with FDR-corrected q-values < 0.2 in both colon tissues (**Figure 3A**).

### 3.4 LA colonization upregulates *Thoc5* expression in GF mice

**Figure 4:**
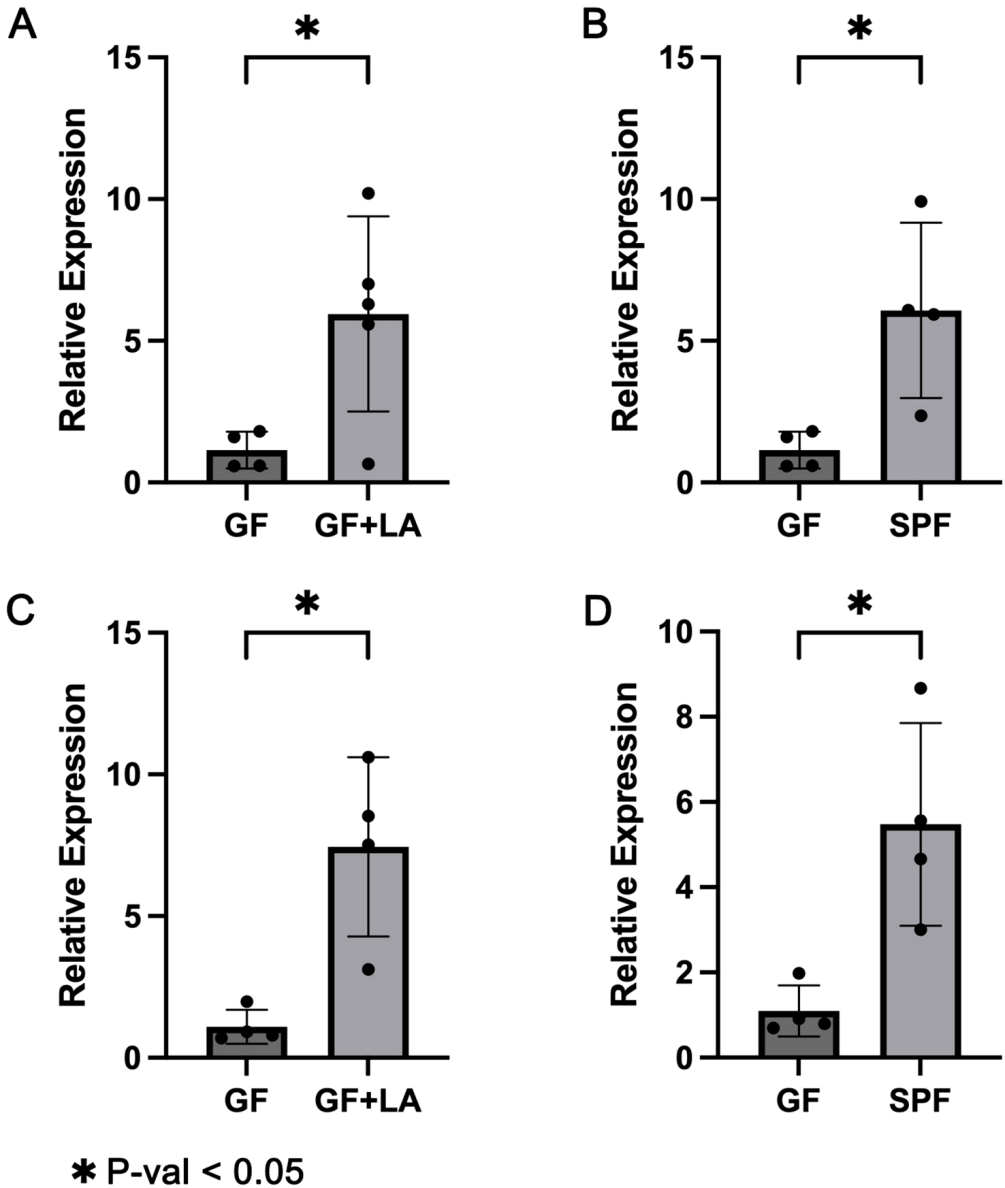
**(A)** RT-qPCR results showing *Thoc5* expression in GF mice before and after LA colonization in Experiment 1. **(B)** RT-qPCR results comparing *Thoc5* expression between GF mice and SPF mice in Experiment 1. **(C)** RT-qPCR results showing *Thoc5* expression in GF mice before and after LA colonization in Experiment 2. **(D)** RT-qPCR results comparing *Thoc5* expression between GF mice and SPF mice in Experiment 2. The symbol * denotes a statistically significant difference (P < 0.05) according to Welch's t-test.

**Figure 5:**
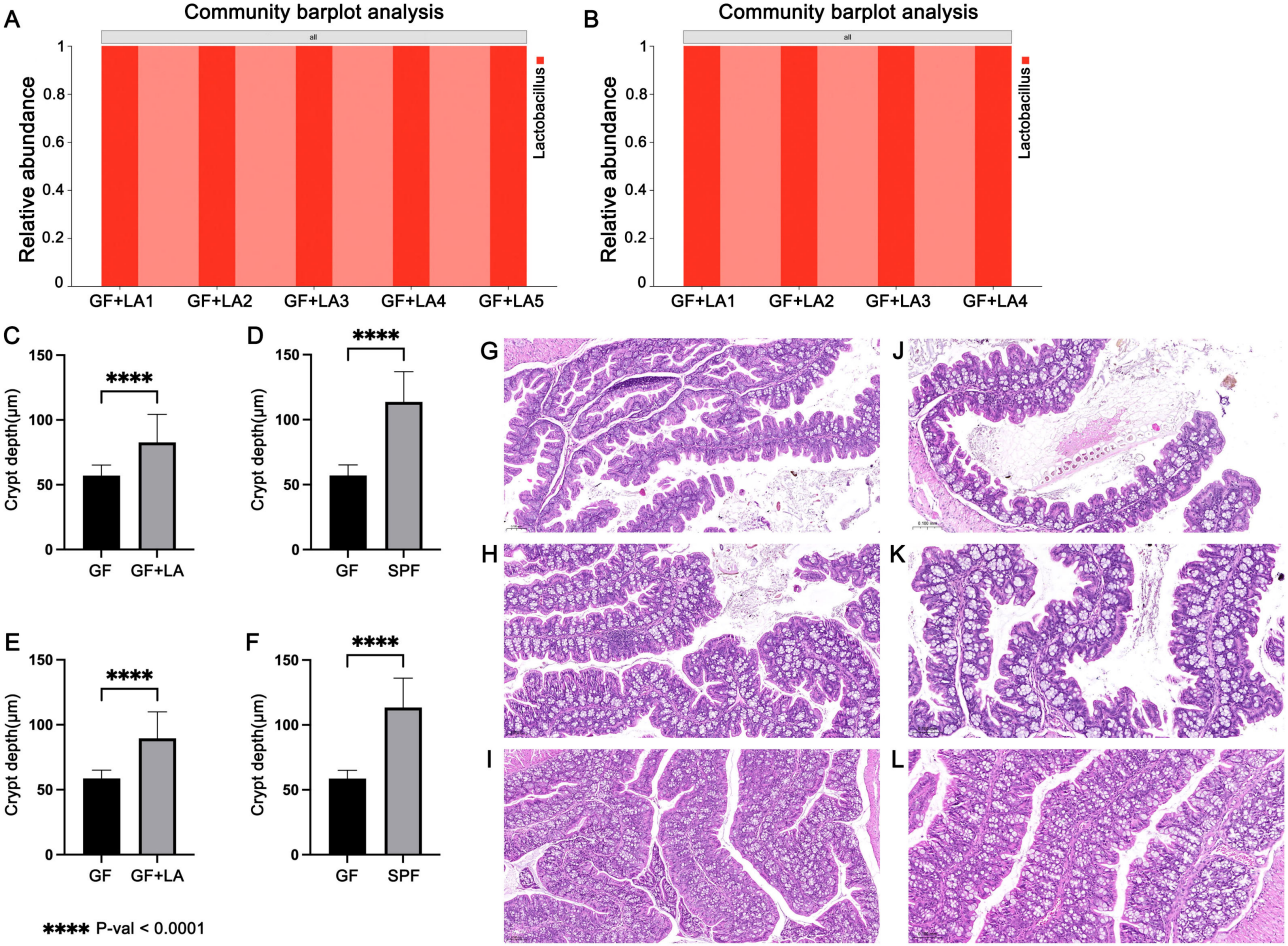
**(A)** 16S rRNA sequencing results of colon contents of four GF mice after LA colonization in Experiment 1. **(B)** 16S rRNA sequencing results of colon contents of four GF mice after LA colonization in Experiment 2. **(C)** Measurement of colonic crypt depth in GF mice before and after LA colonization in Experiment 1. **(D)** Comparison of colonic crypt depth between GF mice and SPF mice in Experiment 1. **(E)** Measurement of colonic crypt depth in GF mice before and after LA colonization in Experiment 2. **(F)** Comparison of colonic crypt depth between GF mice and SPF mice in Experiment 2. **(G)** Representative H&E-stained longitudinal sections of colonic tissues from GF mice in Experiment 1. **(H)** Representative H&E-stained longitudinal sections of colonic tissues from GF mice after LA colonization in Experiment 1. **(I)** Representative H&E-stained longitudinal sections of colonic tissues from SPF mice in Experiment 1. **(J)** Representative H&E-stained longitudinal sections of colonic tissues from GF mice in Experiment 2. **(K)** Representative H&E-stained longitudinal sections of colonic tissues from GF mice after LA colonization in Experiment 2. **(L)** Representative H&E-stained longitudinal sections of colonic tissues from SPF mice in Experiment 2. The symbol **** denotes a statistically significant difference (P < 0.0001) according to Welch's t-test.

To assess whether THOC5 expression is functionally involved in the LA–mediated defence against CDI, we carried out two independent experiments. GF mice were gavaged with live LA strains obtained from two different sources to achieve intestinal colonisation. Throughout housing and handling, no animals exhibited signs of distress or abnormal behaviour: there was no kyphosis, lethargy, reluctance to move, or motor impairment. Fur remained smooth and well groomed, respiration was normal, and no ocular, nasal, or oral discharge was observed. In Experiment 1, GF mice were colonised with LA strain ATCC 4356. RT-qPCR analysis of colon tissues revealed a significant upregulation of *Thoc5* expression in LA-colonized GF mice compared to controls (fold change [FC] = 5.19, P = 0.034; **Figure 4A**). Similarly, *Thoc5* expression differed markedly between GF and SPF mice (FC = 5.30, P = 0.047; **Figure 4B**). Experiment 2 produced concordant results: colonic *Thoc5* mRNA remained significantly up-regulated after LA colonisation (fold change = 6.79, P = 0.025; **Figure 4C**), and levels in the LA-treated GF mice were still markedly higher than in SPF controls (fold change = 5.00, P = 0.031; **Figure 4D**). To verify successful engraftment, all colonic samples were subjected to H&E staining and crypt-depth measurement. GF intestines typically display transient crypt hyperplasia within the first 16 days after exposure to an external microbiota, reflecting increased epithelial proliferation (El Aidy et al., 2012). In both experiments, crypts were significantly deeper following LA colonisation, confirming effective microbial establishment (**Figures 5C–L**). 16S rRNA sequencing of luminal contents from LA-colonized mice confirmed exclusive dominance of LA, ruling out contamination by other bacterial species (**Figures 5A, B**).

### 3.5 Colonic macrophage differentiation in GF mice after colonization

**Figure 6:**
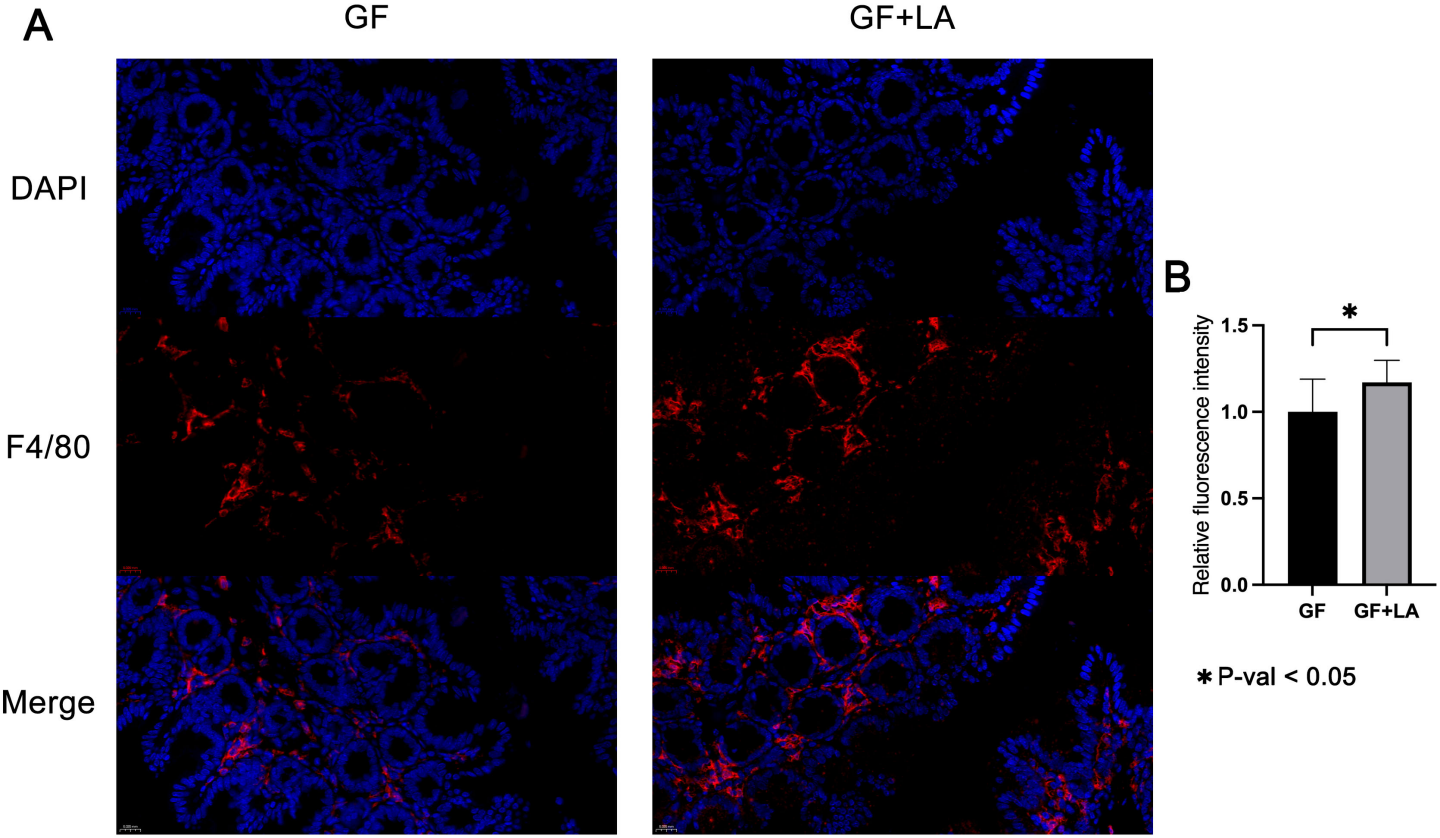
**(A)** Representative longitudinal sections of F4/80 and DAPI immunofluorescence staining in colon tissue from GF and GF+LA mice. **(B)** Quantitative analysis of immunofluorescence density in the colon of GF and GF+LA mice. The symbol * denotes a statistically significant difference (P < 0.05) according to Welch's t-test.

Most work on THOC5 has centred on its role in monocyte-to-macrophage differentiation and maturation (Carney et al., 2009; Tran et al., 2013; Tran et al., 2014). We therefore hypothesised that the LA–induced increase in colonic *Thoc5* expression might drive macrophage differentiation in the gut. F4/80 is a mouse-specific marker of colonic macrophages; its abundance reflects both differentiation status and the extent of macrophage infiltration (Deng et al., 2024; Liu et al., 2025). Accordingly, we stained colonic sections for F4/80 by immunofluorescence and quantified the signal intensity. LA colonisation markedly increased F4/80 expression in the colon of GF mice (fold change = 1.17, P = 0.039; **Figure 6**), indicating enhanced macrophage differentiation and infiltration in response to the probiotic treatment.

## 4 Discussion

Over the past decades, gut microbiota research has emerged as a focal point across diverse disease fields, with well-established roles in modulating host immunity and metabolism (Chung et al., 2012; Holscher, 2017; Adak and Khan, 2019). Although CDI has long been associated with gut dysbiosis (Loo et al., 2011; Brown et al., 2013; Slimings and Riley, 2014; Francino, 2015), prior studies primarily relied on observational approaches with limited sample sizes, leading to inconsistent findings (Martinez et al., 2022) and scant exploration of microbiota-driven host immune regulation. In the present study we combined multi-tissue eQTL data with CDI GWAS results via SMR and identified expression profiles associated with CDI susceptibility. Several genes mapped to the MHC locus, reinforcing the centrality of host antigen-presentation capacity. These findings echo a recent integrative GWAS that linked a CDI-associated variant to HLA-C (Cushing-Damm Kelly et al., 2024). By incorporating transcriptomic instruments, SMR supplies stronger causal inference than GWAS alone, and our multi-tissue approach enhanced sensitivity. Bayesian co-localisation singled out THOC5 as the only gene sharing causal variants with CDI, bolstering confidence in its relevance. Complementary animal experiments confirmed that intestinal LA colonisation up-regulates colonic *Thoc5* and may promote macrophage activation, collectively reducing CDI susceptibility. As a host to trillions of bacteria, fungi, and other microbes, the gut microbiota is often termed the “second genome,” harbouring nearly 100-fold more genes than the human genome (Nelson et al., 2010). This microbial coding potential facilitates immune priming (Kamada et al., 2013) and enhances intestinal barrier integrity (Leclercq et al., 2014). Our findings highlight the MHC region as a critical locus for CDI-associated host genes. MHC genes orchestrate antigen presentation and T-cell activation ​ (Al Naqbi et al., 2021), with established roles in immune disease susceptibility (Fernando et al., 2008). Mounting evidence suggests gut microbiota modulate MHC class I/II gene expression in intestinal tissues (El Aidy et al., 2012; Koyama et al., 2019; Tuganbaev et al., 2020), positioning MHC-driven antigen presentation and immune cell activation as pivotal mechanisms in microbiota-mediated CDI defence. However, few studies have delineated specific microbial species or pathways regulating intestinal MHC expression. For instance, Y. Grace Cao et al. demonstrated that *Faecalibaculum rodentium* enhances MHC class II expression in intestinal epithelial cells via a retinoic acid–eosinophil–interferon-γ axis (Cao et al., 2022).

THOC5 encodes the THO complex subunit 5 protein, a component of the mRNA export machinery (Tran et al., 2014). Current research emphasizes its role in monocyte-macrophage lineage development: THOC5 potentiates receptor signalling to transcription factor activation, promotes monocyte differentiation, and suppresses apoptosis via PI3K-AKT pathway modulation through elevated PIP3 levels (Pierce et al., 2008; Carney et al., 2009). These processes are critical for macrophage and monocyte-derived cell (e.g., osteoclast) differentiation (Tran et al., 2013; Mun et al., 2022). Our study identifies THOC5 as a potential target for LA-driven CDI resistance. Based on prior functional studies, LA colonization may enhance CDI defence by fostering monocyte-macrophage differentiation, a process intrinsically linked to MHC molecule expression—a hallmark of macrophage maturation that underpins pathogen antigen recognition and presentation (Douillard et al., 2021). Most of the genes found in SMR analysis are from the MHC region in chromosome 6. This aligns with our hypothesis that LA colonization bolsters monocyte-macrophage lineage development, thereby strengthening anti-CDI immunity. Our IF analyses corroborated this hypothesis, showing enhanced differentiation and infiltration of colonic macrophages after LA colonisation. Interestingly, *Thoc5* expression was also higher in SPF than in GF mice, possibly reflecting endogenous LA—approximately 10% of the normal murine *Lactobacillus* population (Wang et al., 2019) —or the influence of other protective taxa such as *Lachnospiraceae* (Tejada et al., 2024). Further colonisation studies are needed to dissect these contributions. Regardless of the underlying cause, the results confirm that the normal gut microbiota, including the probiotic species it harbours, plays an indispensable role in maintaining host intestinal health.

While SMR analysis offers robust causal inference, this study has limitations. First, the reliance on European-dominant eQTL and GWAS datasets restricts generalizability to non-European populations. Second, the absence of individual-level data precluded stratified analyses. The study employed germ-free (GF) mice, a choice that both enabled mono-colonisation with a single bacterial species—thus eliminating interference from other microbes—and mimicked the microbiota-depleted state produced by broad-spectrum antibiotics in patients before CDI onset (Soavelomandroso et al., 2017). However, we did not investigate whether further enrichment of Lactobacillus acidophilus in the gut of SPF mice would exert an additional enhancing effect on *Thoc5* expression. Moreover, the absence of single-cell transcriptomics prevented allocation of THOC5 up-regulation to specific cell types. Whether LA influences THOC5 primarily in macrophages, epithelial cells, or both remains unresolved. Future studies using single-cell RNA-seq and macrophage-specific *Thoc5* gain- or loss-of-function mouse models are warranted.

## 5 Conclusion

In summary, this study identifies 14 genes associated with CDI susceptibility using summary-data-based Mendelian randomization (SMR) and confirmed, by Bayesian co-localisation, a strong association between THOC5 expression and CDI susceptibility, identifying THOC5 as a potential host target for prevention. Animal experiments showed that LA colonisation in GF mice up-regulates *Thoc5* and may promote macrophage activation. Our findings underscore the importance of maintaining gut microbial equilibrium and highlight the potential mechanisms by which probiotic microbes interact with host immunity to defend against enteric infections. These results unveil novel therapeutic targets linked to CDI susceptibility, providing innovative insights for future preventive and therapeutic strategies against CDI.

## Data Availability

The datasets presented in this study can be found in online repositories. The names of the repository/repositories and accession number(s) can be found in the article/**Supplementary Material**.

## Supplementary material

Supplementary Table 1 Comprehensive results of SMR analysis integrating eQTL data from blood, transverse colon, and sigmoid colon with CDI.
