# Supplementary material for: The host genes influencing Clostridioides difficile infection and the potential role of intestinal Lactobacillus acidophilus: a Mendelian randomization and animal model study
Source: Front Cell Infect Microbiol. 2025 Jul 8;15:1607476. doi: 10.3389/fcimb.2025.1607476 (PMC12279842; doi:10.3389/fcimb.2025.1607476)

Supplementary Fig. 1

Bayesian co-localisation of the AGER cis-eQTL signal with the CDI GWAS locus, PP.H4.AGER = 1.01e-07.

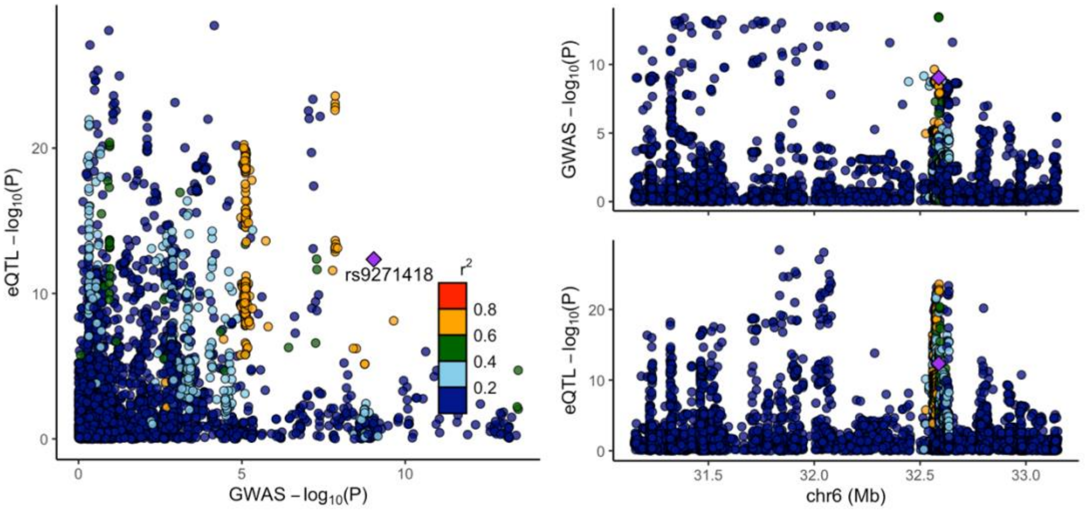

Supplementary Fig. 2

Bayesian co-localisation of the PSMB9 cis-eQTL signal with the CDI GWAS locus, PP.H4.PSMB9 = 1.84e-06.

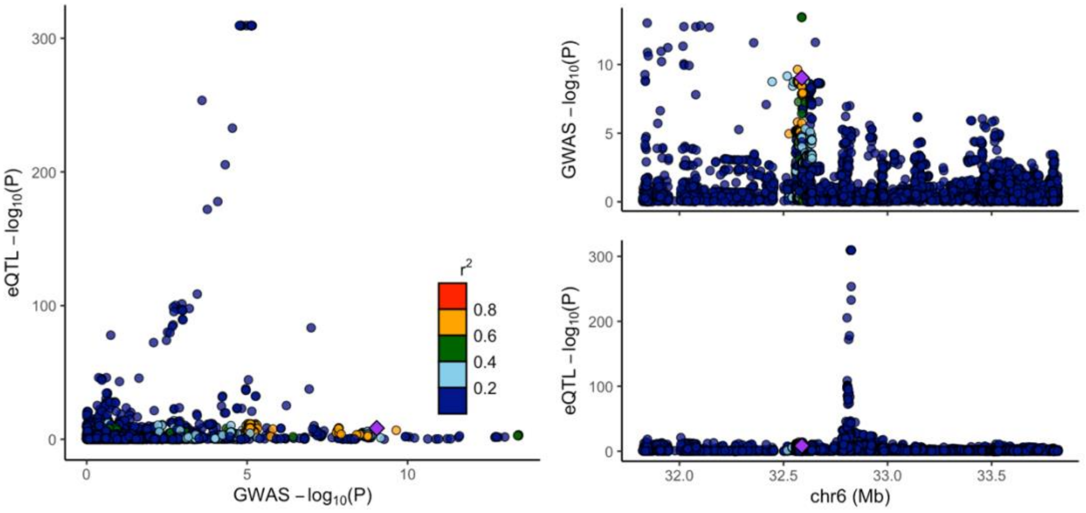

Supplementary Fig. 3

Bayesian co-localisation of the C4A cis-eQTL signal with the CDI GWAS locus, PP.H4.C4A = 5.03e-08.

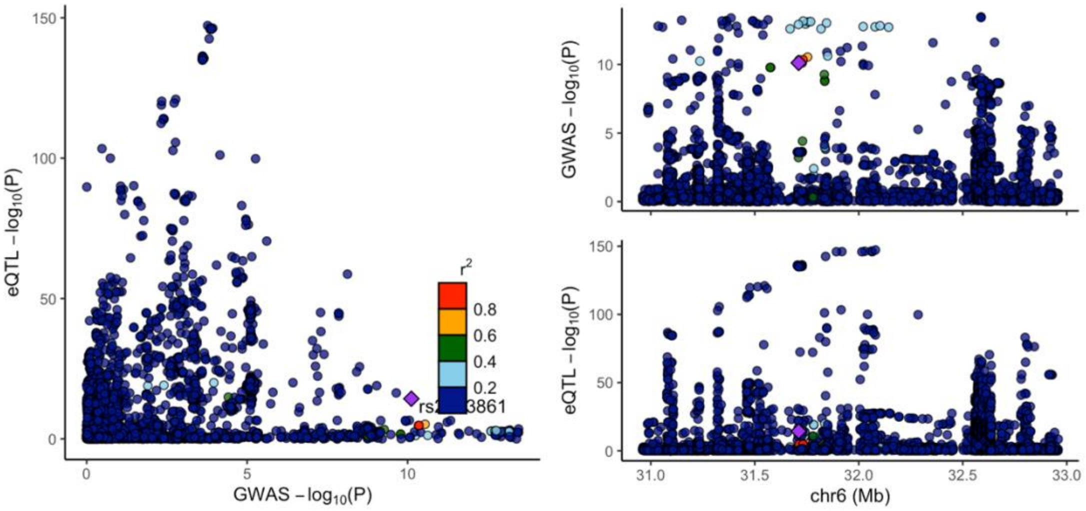

Supplementary Fig. 4

Bayesian co-localisation of the HLA-DQA1 cis-eQTL signal with the CDI GWAS locus, PP.H4.HLA-DQA1 = 5.65e-06.

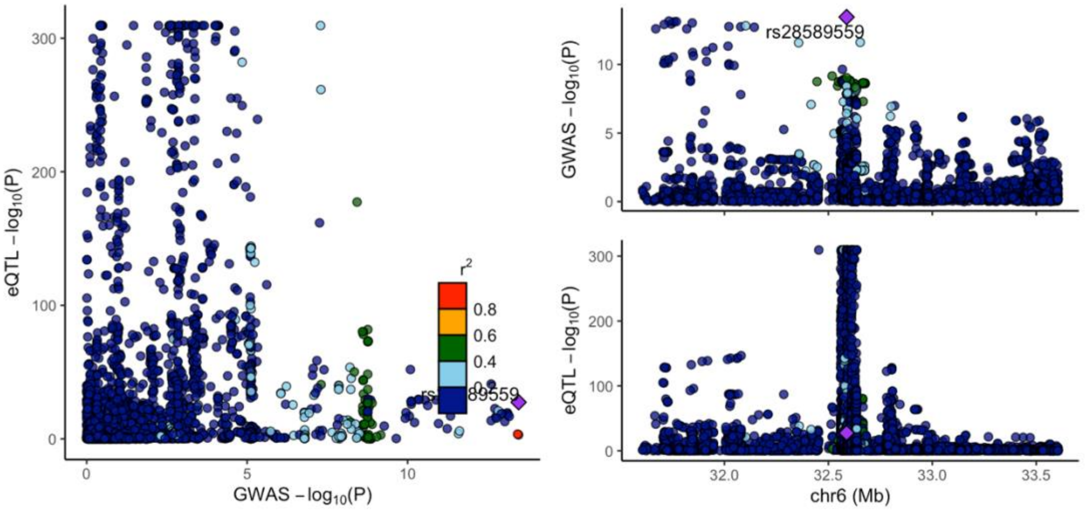

Supplementary Fig. 5

Bayesian co-localisation of the HLA-DQB1 cis-eQTL signal with the CDI GWAS locus, PP.H4.HLA-DQB1 = 8.52e-05.

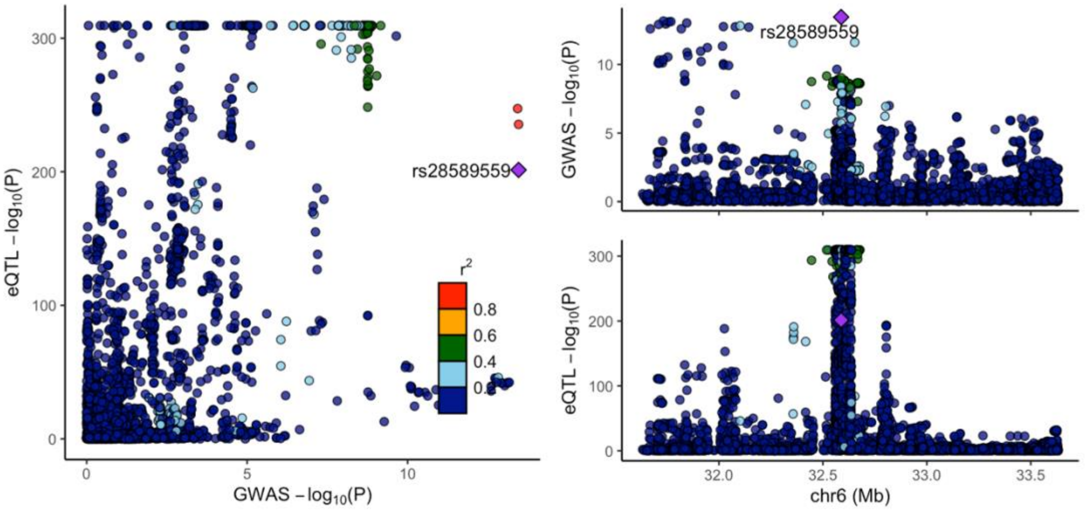

Supplementary Fig. 6

Bayesian co-localisation of the HLA-C cis-eQTL signal with the CDI GWAS locus, PP.H4.HLA-C = 1.34e-06.

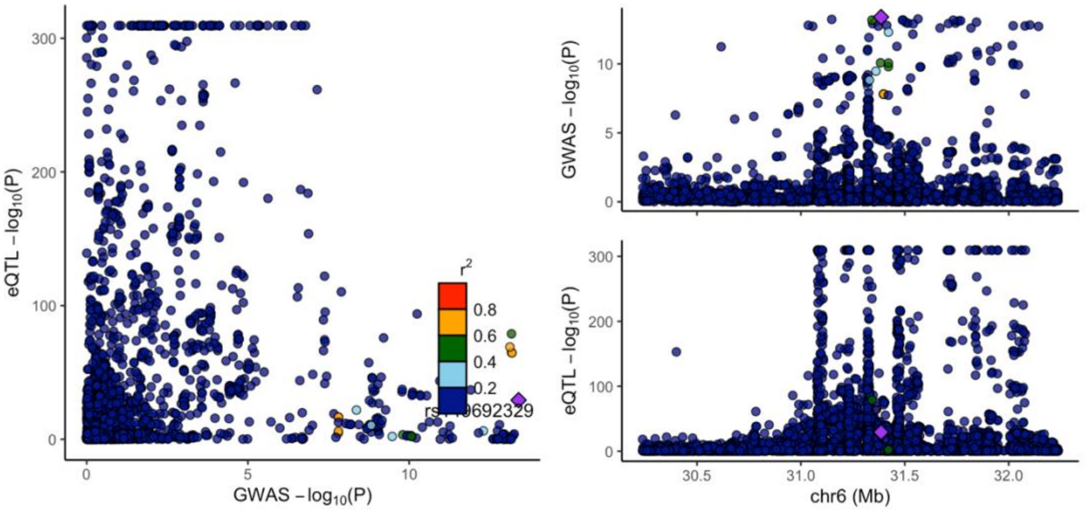

Supplementary Fig. 7
Bayesian co-localisation of the Y_RNA cis-eQTL signal with the CDI GWAS locus, PP.H4.Y_RNA = 1.09e-02.

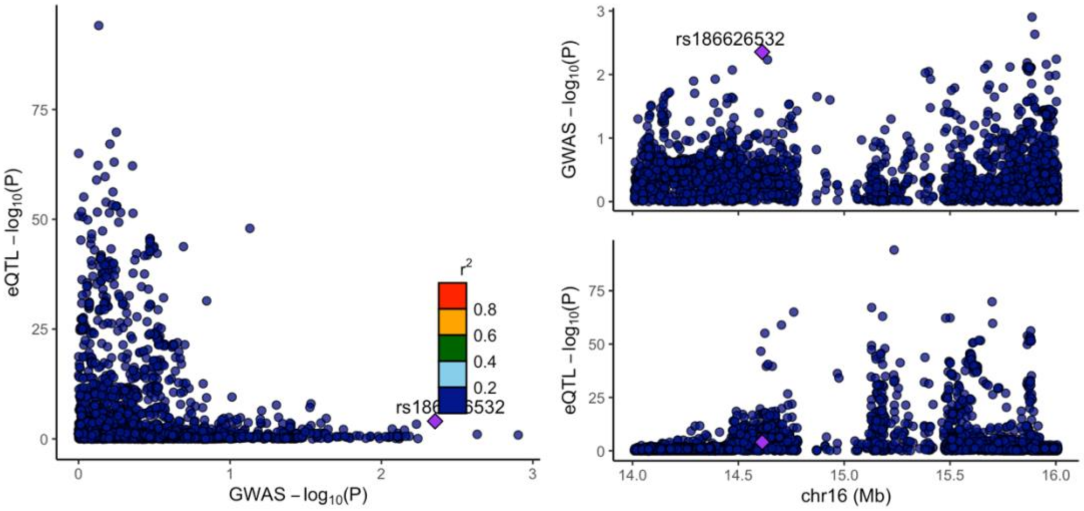

Supplementary Fig. 8
Bayesian co-localisation of the RNF5 cis-eQTL signal with the CDI GWAS locus, PP.H4.RNF5 = 2.29e-09.

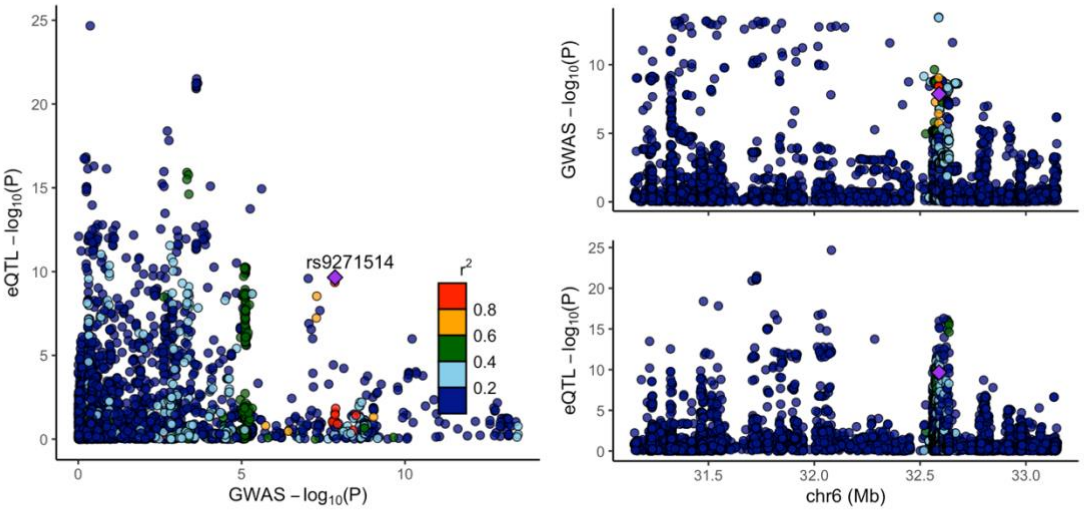

Supplementary Fig. 9

Bayesian co-localisation of the HLA-DRB2 cis-eQTL signal with the CDI GWAS locus, PP.H4.HLA-DRB2 = 2.72e-08.

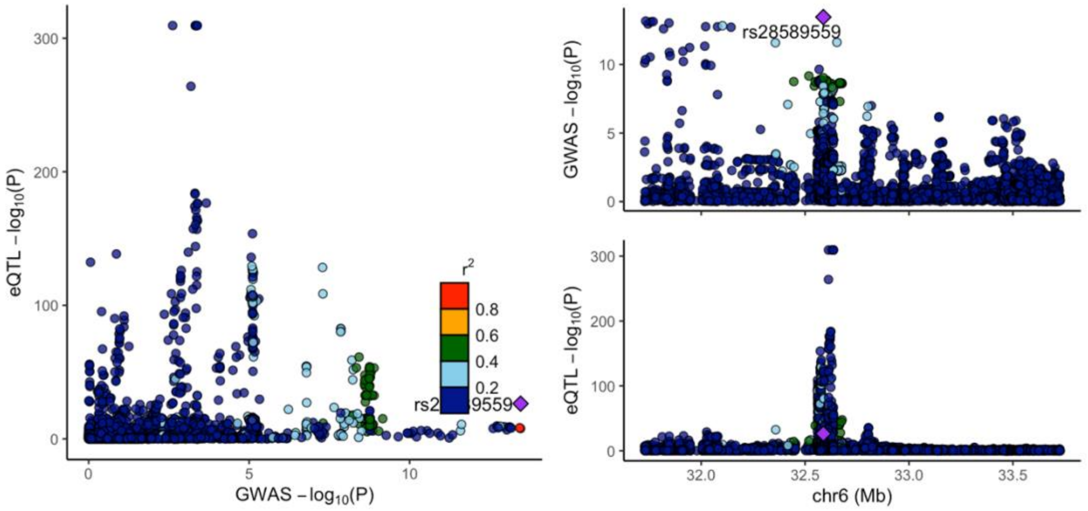

Supplementary Fig. 10

Bayesian co-localisation of the VARS2 cis-eQTL signal with the CDI GWAS locus, PP.H4.VARS2 = 6.55e-06.

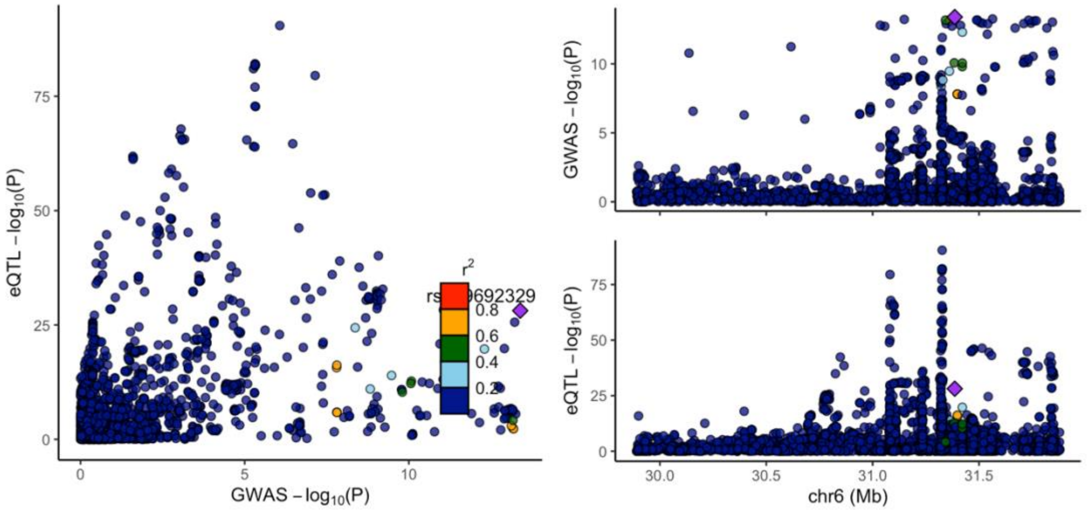

Supplementary Fig. 11

A: Visualization of GO analysis for 12 genes with q-value <0.2, selecting the most significantly enriched 10 terms for BP (Biological Process), CC (Cellular Component), MF (Molecular Function), each term ordered from top to bottom by decreasing q-value.

B: Visualization of KEGG pathway analysis for 12 genes with q-value <0.2, selecting the most significantly enriched 20 pathways, each pathway ordered from top to bottom by decreasing q-value.

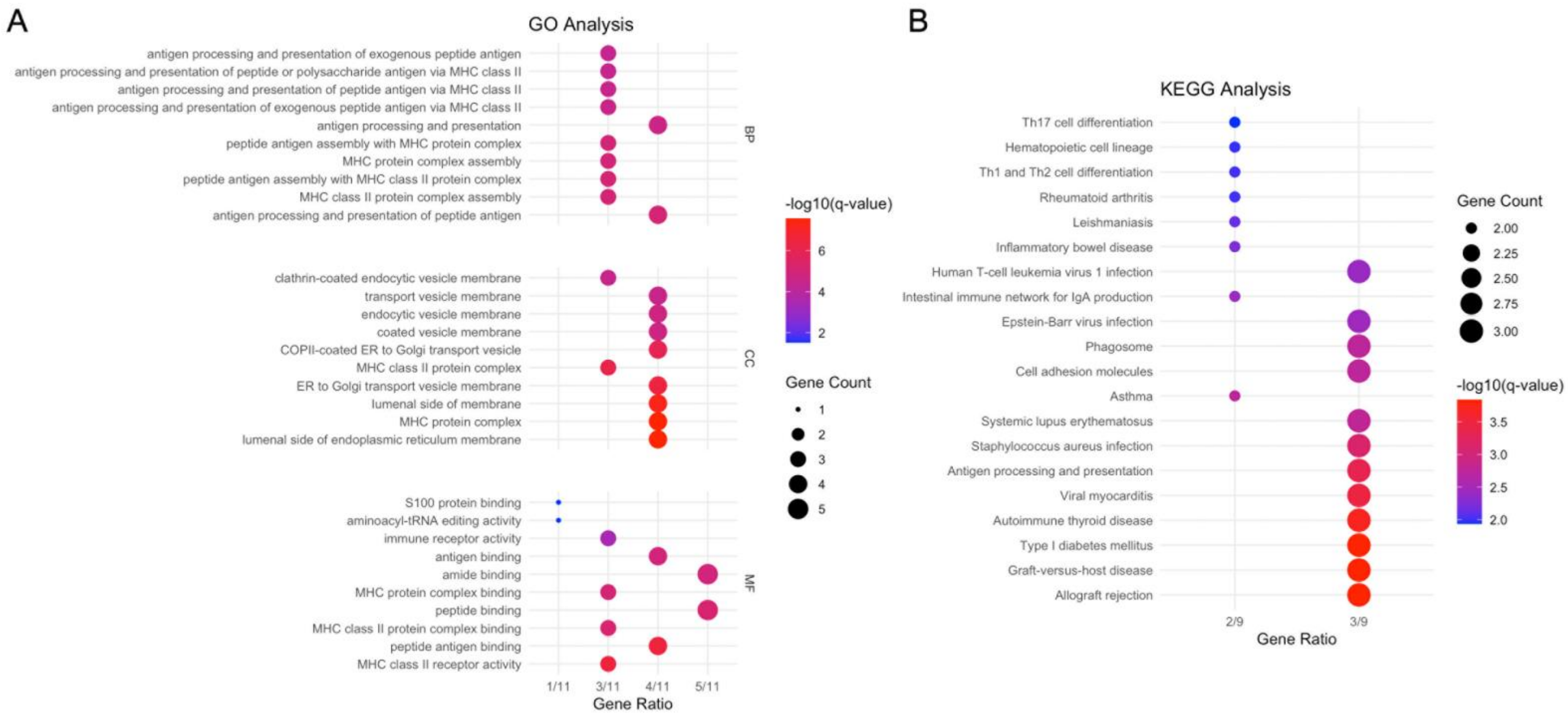

Supplement: Supplementary file 2 [file DataSheet1.pdf]
